# The Impact of Expanded National Health Insurance Coverage of Dentures and Dental Implants on Dental Care Utilization among Older Adults in South Korea: A Study Based on the Korean Health Panel Survey

**DOI:** 10.3390/ijerph17176417

**Published:** 2020-09-03

**Authors:** Jin-Sun Choi, Se-Hwan Jung

**Affiliations:** Department of Preventive and Public Health Dentistry, College of Dentistry, Gangneung-Wonju National University & Research Institute of Oral Science, Gangneung-Wonju National University, Gangneung-si 25457, Korea; jjcjsa@gmail.com

**Keywords:** dental care utilization, dental implant, denture, older adults, insurance coverage

## Abstract

In South Korea, the National Health Insurance Service (NHIS) began its coverage of dentures and dental implants for older people in 2012 and 2014, respectively. This study aimed to investigate the impact of these policies on dental care utilization among people aged 65 years or older according to their sociodemographic characteristics. Data were collected from the Korea Health Panel Survey (KHP; years 2012 and 2015). The statistical significance of the relationships between sociodemographic characteristics and the use of outpatient dental care, denture, and dental implant were analyzed. Results showed an increase of 5.7%, 1.4%, and 2.8% in the use of outpatient dental care, denture, and dental implant, respectively, over the course of three years. Including dentures increased its use by 2.5–3.7 times among people aged 70 years or older. Including dental implants alleviated the disparities among older adults based on age groups and duration of education, except those among uneducated people; however, it caused inequity according to household income. Some Korean older adults remain neglected from the benefits of the expanded NHIS. Therefore, older adults’ access to dental care should be enhanced by the implementation of policies to promote oral health care utilization, dental prosthetic services, and older adults’ insurance coverage.

## 1. Introduction

With the rapid extension of the human lifespan in the late 20th century, interest in older people’s health and quality of life (QOL) is mounting. Diminished masticatory abilities as a result of tooth loss among older people may have an impact on physical functions other than those related to their oral health [1]. Recently, multiple tooth loss among older people has been associated with reduced QOL [2,3,4] and cognitive impairment (e.g., dementia) [5,6,7], thus attracting more attention.

Multiple tooth loss in older people is the cumulative result of dental caries and periodontal diseases that have occurred throughout their lives [8,9]. To restore oral functions hindered by multiple tooth loss, costly dental prosthetic services, such as dentures and dental implants, are needed. Therefore, to prevent multiple tooth loss, the importance of lifelong prevention and management of oral diseases should be emphasized [10,11].

Dental prosthetic services (i.e., dentures and dental implants) are considered as the minimal measures needed to improve oral functions and QOL in older adults with multiple tooth loss. However, utilizing dental prosthetic services is often difficult owing to their cost. The rationale behind this difficulty becomes clear when it is taken into account that multiple tooth loss more frequently affects the socially disadvantaged older people [12]. To resolve such socio-economic issues, government-led dental care projects, or even the expansion of the National Health Insurance (NHI) coverage should be considered.

Korea’s NHI is a system that provides health insurance to all citizens. People who are covered by NHI pay premiums according to their income levels. The National Health Insurance Corporation (NHIC), maintains this premium and provides some insurance premiums when needed [13]. The cost of treatment paid by the people is called an out-of-pocket (OOP) cost. In addition, dentists who provide treatment receive an OOP amount from the patient and insurance premiums from the NHIC [13]. Korean dental health insurance items include X-ray taking, endodontic treatment, dental sealant, composite resin filling, dentures and dental implants for the elderly, and teeth extraction, etc. However, there is a target age standard depending on the type of service [14].

In Korea, the percentage of older adults (≥65 years) in the general population was 7.2% in 2000 and 14.3% in 2018, and it is projected that Korea will become a super-aged society by 2026, with this percentage exceeding 20%; further, the relative poverty rate of Korean older adults (46%) is ranked at the top among the Organisation for Economic Co-operation and Development (OECD) countries, and it is about four times higher than the OECD average of 12.5% [15]. Hence, faced with such a fast-aging rate and the serious issue of poverty among older adults, the Korean government has been actively implementing countermeasures, such as enacting the Framework Act on Low Birthrate in an Aging Society and installing the Presidential Committee on Aging Society and Population Policy in 2005 [16].

In 2015, the percentage of edentulous patients among Korean older adults was 9.2%–0.9% rise from the previous year [17]. Further, 42.9% of Korean older adults in 2017 were reported to have some sort of masticatory discomfort [18]. However, despite the gravity of the problems related to multiple tooth loss among Korean older people, they frequently fail to receive essential dental care owing to financial and policy reasons. In 2011, 56.6% of elderly people aged 65 or older failed to seek treatment (despite believing they needed it) due to financial difficulties. In 2012, it increased to 59.6%. In other words, more than half of the elderly were unable to receive dental treatment due to financial difficulties [19]. Thus, a consensus was reached in Korea toward the need to cover dental prosthetic services under the NHI coverage for older adults having multiple tooth loss. Subsequently, the presidential candidate who pledged to expand the NHI coverage for dentures for older adults was elected in 1997, and, in 2012, the presidential candidate who pledged to expand the NHI coverage for dental implants for older adults was also elected. With this, the NHI then began to cover dentures and dental implants for older adults from July 2012 and July 2014, respectively.

The NHI coverage of dentures was initiated for people aged 75 years or older in July 2012 with an out-of-pocket (OOP) percentage of 30%; the eligibility was expanded to people aged 70 years or older in July 2015 and to people aged 65 years or older in July 2016 [20]. The NHI coverage of dental implants was initiated for people aged 75 years or older in July 2014 for two dentures in a lifetime with an OOP percentage of 50%; the eligibility was expanded to people aged 70 years or older in July 2015 and to people aged 65 years or older in July 2016 [21]. Furthermore, the OOP percentage was reduced from 50% to 30% in July 2018 [22].

No other country around the world provides NHI coverage of dentures and dental implants for older people as Korea currently does. Considering that dental prosthetic services are costly, unlike the internationally recommended preventive dental care services, prompt assessment of this measure undertaken by the Korean government is needed from various angles. Therefore, in this study, data were included from the Korea Medical Panel Survey from the year 2012, when the government introduced the NHI coverage of dental prosthesis services for the elderly, and 2015, three years after the introduction of the service. As a part of this assessment pertaining to the NHI coverage of dentures and dental implants in South Korea, this study aimed to investigate its impact on the dental care utilization of South Korean older people according to their sociodemographic characteristics.

## 2. Materials and Methods

### 2.1. Study Participants

This study utilized secondary data from the Korea Health Panel (KHP). The KHP was developed in 2008 to collect information about Koreans’ medical utilization patterns, medical expenditures, and to analyze the factors that affect these in a comprehensive and in-depth manner [23]. We collected raw data from the KHP for the years 2012 and 2015. To collect the data from the KHP website, we followed the delineated process within the website and conducted an analysis of people aged 65 years or older. A stratified two-stage cluster sampling with probability proportional to size was used, where the sample enumeration district clusters were extracted in the first stage, the sample households were selected from the clustered districts in the second stage, and then the members of the sample households were surveyed. In 2008, the original sample consisted of 24,616 people from 7866 households, while 17,417 people from 5850 households and 14,344 people from 5098 households were surveyed in 2012 and 2015, respectively. In 2012, new samples (7387 people from 2520 households) were then added to the original sample. And, 2983 people in 2012 and 4044 people in 2015 were used in the final analysis for older adults aged 65 and above [24]. The KHP data were reviewed by the institutional review board at the Korea Institute for Health and Social Affairs.

In this study, we selected the annual use of outpatient dental care, denture use, and dental implant use as the dependent variables; for the sociodemographic characteristics, we included sex, age group, marital status, duration of education, household income, and type of health insurance. Age groups were classified into 65–69 years, 70–74 years, 75–79 years, and ≥80 years, while marital status was classified into married and not married (comprising separated, widowed, missing, divorced, never married). Duration of education was classified into 0, 1–6, 7–12, and ≥13 years, while household income was classified into 1st quintile (lowest) to 5th quintile (highest). Type of health insurance was classified into NHI and medical care, wherein the latter is a system that guarantees help for the medical problems of low-income groups who cannot maintain their lives or sustain a living [25].

### 2.2. Statistical Analysis

Statistical analyses were conducted using the STATA 13.0 (Stata Statistical Software: Release 13. College Station, TX: StataCorp LP, USA). The statistical significance in the relationships between sociodemographic characteristics and the use of outpatient dental care, denture, and dental implant by year was analyzed with chi-square tests. And respondents with incomplete responses were excluded from the analysis. Further, the association between dental care utilization and modified sociodemographic characteristics was analyzed by year using a multiple logistic regression model to compute odds ratio and 95% confidence levels. Estimated values were calculated by applying the cross-sectional weights, and the number of samples (N) was presented as unweighted values.

## 3. Results

As a result of the expanding coverage of dental prostheses for older people, dental care utilization, denture use, and dental implant use all increased in 2015 compared to their use in 2012, with an increase of 5.7% (24.7 to 30.4), 1.4% (3.5 to 4.9), and 2.8% (2.3 to 5.1), respectively. The changes in the annual dental care utilization according to sociodemographic characteristics are shown in Table 1.

Overall, dental care utilization was higher among men, younger age groups, married people, people with a higher duration of education, higher household income, and those with NHI. In 2012, only the age group and duration of education were statistically significant (*p* < 0.001). However, in 2015, dental care utilization significantly increased with sociodemographic characteristics (*p* < 0.01, *p* < 0.001).

In 2012, denture use did not significantly differ according to any of the sociodemographic characteristics. However, in 2015, denture use significantly increased among people aged 70 years or older and the unmarried (*p* < 0.001). Additionally, the trend towards increased denture use was strengthened among women, people with a lesser duration of education, lesser household income, and those receiving medical care. However, the results for these were not statistically significant.

Dental implant use was higher among men, younger age groups, married people, people with a higher duration of education, higher household income, and those with health insurance. In 2012, it was significant with age group, duration of education, and household income (*p* < 0.001, *p* < 0.01, *p* < 0.05, respectively). In 2015, it was significant with gender (*p* < 0.01), in addition to the variables with which it was significant in 2012.

After coverage expansion, a greater number of sociodemographic characteristics significantly differed in outpatient dental utilization and denture use. Meanwhile, although dental implant use was clearly associated with household income, it had weaker associations with other characteristics (Table 2).

The ORs(Odds Ratio) between duration of education and dental care utilization in 2012 were found to be 0.84, 0.60, and 0.42 for the three age groups of 7–12 years, 1–6 years, and 0 years, respectively; in 2015 they were 0.82, 0.64, and 0.39, respectively. The inequality gap between educational levels was maintained and statistically significant. The ORs of household income in 2012 were 0.82 (highest), 0.83, 0.82, and 0.87 (lowest) for the 4th, 3rd, 2nd, and 1st quintiles, respectively; in 2015 they were 0.75, 0.84, 0.61, and 0.75, respectively, with a slight increase in the gap. In particular, the value for the 2nd quintile decreased from 0.8 to 0.6 and was statistically significant.

The ORs between age group and denture use in 2012 were found to be 0.76, 0.99, and 0.55 for the categories 70–74 years, 75–79 years, and ≥80 years of age, respectively; in 2015, they were 2.54, 3.69, and 2.73, respectively. Their statistical significance was also confirmed. In addition, the ORs for duration of education, household income, and type of health insurance decreased slightly in 2015 compared to 2012 but were not statistically significant.

The ORs between dental implant use and duration of education in 2012 were found to be 0.31, 0.36, and 0.26 for the categories 7–12 years, 1–6 years, and 0 years, respectively; in 2015 they were 0.69, 0.73, and 0.21, respectively. This shows that the gap between the duration of education increased, and the statistical significance of 0 years (lowest) was maintained. In addition, the ORs for household income in 2012 were found to be 0.54 (highest), 0.63, 0.83, and 0.40 (lowest) for the 4th, 3rd, 2nd, and 1st quintiles, respectively; in 2015 they were 0.62, 0.54, 0.44, and 0.37, respectively. This shows a slight but statistically significant increase in the income gap. 

## 4. Discussion

The importance of lifelong prevention and management of dental caries and periodontal diseases to address the problem of multiple tooth loss in older people has been highlighted. Therefore, the Korean government initiated the expansion of the NHI coverage to provide dental prosthetic services for older people, mainly because there was a social consensus among the Korean population regarding its rapidly aging society and the serious problem of poverty among the Korean elderly. However, such expansion has not been attempted elsewhere in the world because dental prosthetic services are costly; hence, this provision may incur financial burden. Thus, we aimed to review the usefulness of this policy by investigating its effects on dental care utilization according to the sociodemographic characteristics of the Korean older people.

First, implementing health insurance coverage of dentures and dental implants for older people in Korea increased the utilization of the aforementioned services and annual outpatient dental care (Table 1). However, over the next three years after implementation (during which insurance coverage was expanded), the increase in rates for outpatient dental utilization, denture use, and dental implant use, as found through our analysis, were only 5.7%, 1.4%, and 2.8%, respectively. This suggests that the policy has only enhanced access to dental care for a particular population as opposed to promoting universal health coverage. This may have been inevitable as there were clear limitations in improving access to dental care by expanding coverage to dental prosthetic services. In 2015, the percentage of Korean older people who needed dentures was 22.7% [26], while it was around 31.5% from 2007 to 2009 [27]. Further, such a low increase may also be attributable to the high coverage cost per dental implant and denture (1.3 million South Korean won or 1126 United States of America dollars [USD] for dentures) [28], which led to various limitations (e.g., age limit, high OOP cost, and a limited number of covered dental implants) in accessing insurance benefits. In fact, the rapid escalation of treatment cost owing to the dental care coverage expansion (about 31.45 million USD in 2012; about 443.67 million USD in 2015) confirms that there are practical limitations for this expansion owing to the consequent financial burden [29].

Meanwhile, the income poverty rate for older adults in Korea was 47.2%, the highest among OECD members, and about 3.7 times higher than the OECD average (12.8%) [30]. The average monthly income of senior citizens in 2017 was 986 USD, 792 USD, 767 USD, and 647 USD for those aged 65–69 years, 70–74 years, 75–79 years, and 80–84 years, respectively [31]. The cost of health insurance dentures and implants is about 1100 USD, thus, the elderly in Korea feel burdened by dentures and implants due to a lack of finances. Therefore, we suggest that the government take a high-risk approach by focusing on mitigating sociodemographic disparities in access to such services as opposed to promoting universal health coverage.

At a time when Korea’s aging population is rapidly progressing, a system that enables prosthetic services to be prioritized for those who absolutely need them will be more effective than strengthening dental prosthetic health insurance for the elderly with weak accessibility. With this, there will be a more efficient use of limited resources. In addition, it is judged that limited resources can be used effectively by strengthening preventive services for young people. Some European countries have implemented preventive policies for children and adolescents under the age of 18 under the Non-Operative Caries Treatment Program (NOCTP) method and have identified positive long-term effects [32,33]. These policies argue that strengthening prevention is preferable to treating diseases [34,35].

Next, logistic regression models revealed varying changes regarding access to dental care according to sociodemographic characteristics following the studied expansion (Table 2).

What is noteworthy in the association between household income and dental care utilization is that the ORs for the 2nd quintile are relatively significant, from 0.8 to 0.6. In fact, the 1st quintile with the lowest income level will receive a remission in its OOP amount, while the 2nd quintile with the lowest income will have to pay its OOP amount. Therefore, it can be estimated that the dental care utilization in the 1st quintile could be relatively increased compared to the 2nd quintile, and that the ORs of dental care utilization in the 2nd quintile was the lowest.

Meanwhile, denture coverage alleviated inequalities in access due to social characteristics (i.e., duration of education, household income, and type of health insurance), but not to a statistically significant extent. One of the main changes was that it increased dental care utilization by 2.5–3.7 times among people aged 70 years or older compared to people aged 65–69 years. A study on Brazilian adults also found that the need for a complete set of dentures increased with age and that it was closely related to an individual’s socioeconomic status [36]. Further, expanded dental implant coverage mitigated gaps among different age groups and people with differing durations of education—except for uneducated people; still, it also induced a clear stepwise pattern of inequality according to household income. The reason for these differences may be that although denture provision is perceived as an essential service that is utilized by people aged 70 years or older when needed, high-income individuals are still more likely to utilize dental implants owing to the different perceptions of OOP cost between low- and high-income individuals. Therefore, this shows that there is a need for a complete review of the policy, mainly because it seems that the dental implant coverage actually worsened access inequality, contradicting its original intent, which was to alleviate sociodemographic inequalities in access to dental services by breaking down financial barriers (e.g., by lowering eligible age and OOP rate).

For this policy review, we suggest referencing the Swedish coverage system, where dental implants are covered only in essential cases. Since the launching of dental insurance in 1974, Sweden has been offering a comprehensive and costly prosthetic care to middle-aged or older people [37], and the Dental Health Insurance Plan implemented in 2002 mentions the provision of subsidies for costly dental prostheses for people aged 65 years or older [38]. Moreover, the scope of dental implant coverage is determined by the number of lost teeth [39]. In contrast, Korean health insurance covers only two dental implants for people aged 65 years or older during their whole lives. Considering that, in 2015, the average number of remaining natural teeth was 17.5 among older adults aged 65 years or older [17], the coverage of only two dental implants is not sufficient for their current oral health status. Therefore, the dental implant coverage policy should focus on restoring their minimal masticatory functions to ensure good QOL as opposed to simply increasing the number of teeth. To this end, policies that take into consideration the oral health status and socioeconomic levels of the target population should be implemented.

Although the annual dental care utilization increased by 5.7% over three years of the expansion, the utilization rate remained at 30.4%, highlighting that most Korean older people still struggle to utilize the national dental care coverage. Universal health coverage refers to the assurance that everyone can receive high-quality essential health services without experiencing financial difficulties [40]. Korea’s NHIS can be universally accessible to all Koreans, but there is still a limit to universal health coverage because of the financial burden of OOP spending. Therefore, even if the government pays for some of the cost by covering dentures and dental implants under health insurance, older people with low income may still face financial limitations due to the presence of OOP spending Korea’s NHIS can be universally accessible to all Koreans, but there is still a limit to universal health coverage because of the financial burden of OOP spending. For example, prosthetics such as dentures and dental implants are expensive avail of. Therefore, even if the government pays for some of the cost by covering dentures and dental implants under health insurance, older people with low income may still face financial limitations due to the presence of OOP spending.

In 2001, the Ministry of Health and Social Care of the United Kingdom published the “National Service Framework for Older People,” and it has aimed to provide fair, high-quality, and comprehensive healthcare and social welfare services to British older people [41]. Fisher et al. suggested including dental insurance as part of health insurance, thus expanding universal health coverage to include oral health [42]. The Japanese government, dealing with an already super-aged society, implemented a medical care policy for the elderly in 1973, in which all medical costs for people aged 70 years or older are paid for by the government [43]. Japan is also planning to complete the provision of a regional care system by 2025, which aims to comprehensively provide health services, health promotion, home care, rehabilitation, and welfare to the entire Japanese older adult population [44]. Borreani et al. suggested the need for individual and systemic change at the societal level to enhance the elderly’s access to care [45]. Taking into account the current situation of older adults’ access to oral health care, and the fact that Korea is facing an aging society that may become similar to that of Japan, we believe that the Korean government may need to adopt Japan’s healthcare delivery system to diminish health disparities among Korean older adults. Most of all, we believe that the NHI should mainly cover preventive care services for the oral health of older adults, rather than providing dental prosthetic services. Furthermore, for older adults with physical or cognitive disabilities who cannot access the dental care policies targeted for them, initiatives that provide home visit services and community care should be explored. For instance, dental care should be made more accessible for older people living in long-term care facilities. In some prior studies, oral health care teams were required to visit long-term care facilities regularly to manage the oral health of the elderly in nursing homes, which resulted in the stabilization of the oral health of a significant proportion of the elderly [46,47]. Therefore, the establishment of a visiting service and a dental care delivery system for the elderly living in these types of facilities should also be developed.

Owing to the cross-sectional nature of this study, we were able to examine the association between dental prosthetic services and sociodemographic factors among older people, but not their causal relationships. In addition, there is the possibility of self-report biases arising from the self-reported responses. Nevertheless, this study is significant in that it investigated the impact of dental prosthetic coverage expansion on dental care utilization of older people according to their sociodemographic characteristics, and also reviewed the usefulness of the said policy. In the future, researchers should make longitudinal examinations to establish the causal relationships between dental prosthetic services and sociodemographic factors among older Korean adults.

## 5. Conclusions

This study found that regardless of the provided expansion in the NHI coverage, socially disadvantaged older adults remain neglected from denture and dental implant services in South Korea. Thus, the national coverage system for preventive oral care and dental prosthetic services should consider older people’s sociodemographic characteristics and promote universal health coverage; further, policies should be developed and implemented toward the provision of home visit services and community care for older people with limited access to dental care.

## Figures and Tables

**Table 1:** Changes in the annual dental care utilization according to sociodemographic characteristics after expanded coverage of dental prostheses for older adults.

Classification	N		Dental Care Utilization						Denture Use						Dental Implant Use					
	2012 Year	2015 Year	2012 Year			2015 Year			2012 Year			2015 Year			2012 Year			2015 Year		
			N	(Weighted%)		N	(Weighted%)		N	(Weighted%)		N	(Weighted%)		N	(Weighted%)		N	(Weighted%)	
**Total**	2983	4044	736	(24.7)		1209	(30.4)		112	(3.5)		204	(4.9)		65	(2.3)		197	(5.1)	
**Gender**																				
Male	1290	1720	329	(25.5)	^NS^	559	(33.1)	**	48	(3.6)	^NS^	81	(4.5)	^NS^	28	(2.4)	^NS^	103	(6.4)	**
Female	1693	2324	407	(24.1)		650	(28.5)		64	(3.5)		123	(5.2)		37	(2.2)		94	(4.2)	
**Age Group**																				
65–69 years	909	1367	271	(29.1)	***	444	(33.4)	***	36	(3.7)	^NS^	30	(2.1)	***	35	(3.8)	***	94	(7.1)	***
70–74 years	990	962	251	(25.7)		336	(35.5)		31	(3.1)		55	(5.4)		21	(2.3)		48	(5.1)	
75–79 years	650	928	156	(24.6)		264	(28.7)		30	(4.4)		74	(7.9)		8	(1.4)		37	(4.2)	
≥80 years	434	787	58	(13.0)		165	(21.6)		15	(3.1)		45	(6.2)		1	(0.2)		18	(2.8)	
**Marital status**																				
Married	2024	2697	520	(25.7)	^NS^	858	(32.2)	**	70	(3.3)	^NS^	115	(4.0)	**	46	(2.4)	^NS^	144	(5.7)	^NS^
Not married	959	1347	216	(22.6)		351	(27.3)		42	(4.0)		89	(6.6)		19	(2.0)		53	(4.2)	
**Duration of education**																				
0 years	494	591	78	(16.2)	***	110	(18.5)	***	12	(2.2)	^NS^	33	(6.2)	^NS^	5	(1.1)	**	9	(1.3)	***
1–6 years	1303	1657	305	(23.2)		460	(28.5)		50	(3.6)		91	(5.2)		27	(2.2)		72	(4.9)	
7–12 years	961	1464	284	(29.5)		510	(34.9)		44	(4.4)		69	(4.6)		21	(2.1)		82	(5.7)	
≥13 years	225	332	69	(31.7)		129	(39.8)		6	(2.7)		11	(3.0)		12	(6.1)		34	(6.7)	
**Household income**																				
1st quintile (lowest)	1168	1703	274	(23.5)	^NS^	465	(28.1)	***	55	(4.4)	^NS^	102	(6.3)	^NS^	13	(1.3)	*	50	(3.1)	***
2nd quintile	766	1084	184	(24.2)		312	(27.9)		28	(3.5)		53	(4.3)		22	(2.9)		51	(4.5)	
3rd quintile	534	566	136	(25.1)		195	(34.8)		15	(2.7)		23	(4.4)		12	(2.5)		36	(6.1)	
4th quintile	311	414	80	(25.5)		128	(32.3)		7	(2.2)		14	(4.0)		9	(2.4)		30	(7.5)	
5th quintile (highest)	200	277	61	(30.0)		109	(39.3)		7	(3.2)		12	(3.3)		9	(4.6)		30	(10.4)	
**Type of health insurance**																				
NHI	2668	3654	654	(24.6)	^NS^	1110	(30.7)	*	92	(3.3)	^NS^	185	(4.8)	^NS^	63	(2.5)	^NS^	188	(5.3)	^NS^
Medical care	265	260	62	(22.3)		54	(22.8)		16	(5.4)		13	(6.2)		1	(0.5)		2	(1.5)	

^NS^*p* > 0.05, * *p* < 0.05, ** *p* < 0.01, *** *p* < 0.001. The data were analyzed by a chi-square test. Incomplete responses were excluded from the analysis. Household income (2012 year: 4), Type of health insurance (2012 year: 50, 2015 year: 130). NHI: the National Health Insurance.

**Table 2:** Changes in the associations between the annual dental care utilization and sociodemographic characteristics after expanded coverage of dental prostheses for older adults.

Classification	Dental Care Utilization		Denture Use		Dental Implant Use	
	2012 Year	2015 Year	2012 Year	2015 Year	2012 Year	2015 Year
	Odds Ratio (95% CI)	Odds Ratio (95% CI)	Odds Ratio (95% CI)	Odds Ratio (95% CI)	Odds Ratio (95% CI)	Odds Ratio (95% CI)
**Gender**						
Male	Ref. 1.000	Ref. 1.000	Ref. 1.000	Ref. 1.000	Ref. 1.000	Ref. 1.000
Female	1.147(0.932–1.412) ^NS^	1.040(0.870–1.243) ^NS^	1.021(0.636–1.638) ^NS^	0.966(0.632–1.474) ^NS^	1.124(0.632–1.999) ^NS^	0.832(0.583–1.187) ^NS^
**Age Group**						
65–69 years	Ref. 1.000	Ref. 1.000	Ref. 1.000	Ref. 1.000	Ref. 1.000	Ref. 1.000
70–74 years	0.891(0.717–1.106) ^NS^	1.250(1.023–1.528) *	0.760(0.454–1.274) ^NS^	2.544(1.440-4.494) **	0.656(0.365–1.181) ^NS^	0.916(0.609–1.377) ^NS^
75–79 years	0.842(0.655–1.083) ^NS^	0.963(0.775–1.195) ^NS^	0.996(0.588–1.687) ^NS^	3.690(2.101–6.483) ***	0.355(0.148–0.849) *	0.817(0.500–1.333) ^NS^
≥80 years	0.389(0.272–0.555) ***	0.666(0.514–0.865) **	0.556(0.263–1.173) ^NS^	2.735(1.452–5.151) **	0.062(0.008–0.490) **	0.556(0.283–1.095) ^NS^
**Marital status**						
Married	Ref. 1.000	Ref. 1.000	Ref. 1.000	Ref. 1.000	Ref. 1.000	Ref. 1.000
Not married	1.138(0.911–1.422) ^NS^	1.089(0.894–1.327) ^NS^	1.495(0.930–2.402) ^NS^	1.548(0.953–2.516) ^NS^	1.491(0.809–2.771) ^NS^	1.206(0.788–1.847) ^NS^
**Duration of education**						
≥13 years	Ref. 1.000	Ref. 1.000	Ref. 1.000	Ref. 1.000	Ref. 1.000	Ref. 1.000
7–12 years	0.848(0.604–1.191) ^NS^	0.821(0.614–1.097) ^NS^	1.699(0.630–4.582) ^NS^	1.254(0.598–2.629) ^NS^	0.315(0.148–0.671) **	0.693(0.427–1.122) ^NS^
1–6 years	0.606(0.427–0.860) **	0.643(0.474–0.871) ***	1.258(0.455–3.481) ^NS^	1.100(0.514–2.352) ^NS^	0.362(0.163–0.806) *	0.739(0.445–1.228) ^NS^
0 years	0.427(0.278–0.657) ***	0.397(0.269–0.585) ***	0.712(0.216–2.352) ^NS^	1.055(0.445–2.497) ^NS^	0.260(0.078–0.865) *	0.219(0.088–0.540) **
**Household income**						
5th quintile (highest)	Ref. 1.000	Ref. 1.000	Ref. 1.000	Ref. 1.000	Ref. 1.000	Ref. 1.000
4th quintile	0.826(0.546–1.250) ^NS^	0.753(0.524–1.802) ^NS^	0.737(0.231–2.350) ^NS^	0.876(0.339–2.263) ^NS^	0.548(0.207–1.451) ^NS^	0.623(0.346–1.121) ^NS^
3rd quintile	0.830(0.567–1.215) ^NS^	0.846(0.603–1.186) ^NS^	0.930(0.346–2.496) ^NS^	0.928(0.415–2.072) ^NS^	0.634(0.246–1.636) ^NS^	0.547(0.314–0.955) *
2nd quintile	0.822(0.569–1.188) ^NS^	0.611(0.446–0.837) **	1.138(0.447–2.897) ^NS^	1.240(0.598–2.569) ^NS^	0.839(0.364–1.931) ^NS^	0.442(0.263–0.743) **
1st quintile (lowest)	0.874(0.610–1.253) ^NS^	0.752(0.553–1.023) ^NS^	1.513(0.620–3.689) ^NS^	1.305(0.658–2.589) ^NS^	0.407(0.160–1.035) ^NS^	0.374(0.204–0.685) **
**Type of health insurance**						
NHI	Ref. 1.000	Ref. 1.000	Ref. 1.000	Ref. 1.000	Ref. 1.000	Ref. 1.000
Medical care	1.027(0.739–1.426) ^NS^	0.785(0.544–1.133) ^NS^	1.436(0.808–2.551) ^NS^	0.948(0.488–1.842) ^NS^	0.291(0.040–2.084) ^NS^	0.424(0.086–2.085) ^NS^

^NS^*p* > 0.05, * *p* < 0.05, ** *p* < 0.01, *** *p* < 0.001. The data were analyzed by a logistic regression.
